# Crystallographic anomalous diffraction data for the experimental phasing of two myelin proteins, gliomedin and periaxin

**DOI:** 10.1016/j.dib.2017.02.049

**Published:** 2017-03-06

**Authors:** Huijong Han, Petri Kursula

**Affiliations:** a Faculty of Biochemistry and Molecular Medicine, University of Oulu, Finland; b Department of Biomedicine, University of Bergen, Norway

**Keywords:** Crystallography, Protein, Anomalous signal, Experimental phasing, Synchrotron

## Abstract

We present datasets that can be used for the experimental phasing of crystal structures of two myelin proteins. The structures were recently described in the articles “Periaxin and AHNAK nucleoprotein 2 form intertwined homodimers through domain swapping” (H. Han, P. Kursula, 2014) [1] and “The olfactomedin domain from gliomedin is a β-propeller with unique structural properties” (H. Han, P. Kursula, 2015) [2]. Crystals of periaxin were derivatized with tungsten and xenon prior to data collection, and diffraction data for these crystals are reported at 3 and 1 wavelengths, respectively. Crystallographic data for two different pressurizing times for xenon are provided. Gliomedin was derivatized with platinum, and data for single-wavelength anomalous dispersion are included. The data can be used to repeat the phasing experiments, to analyze heavy atom binding sites in proteins, as well as to optimize future derivatization experiments of protein crystals with these and other heavy-atom compounds.

**Specifications Table**

**Table:** 

Subject area	*Biology*
More specific subject area	*Structural biology*
Type of data	*Diffraction datasets, graphs, tables*
How data was acquired	*Synchrotron X-ray data collection*
Data format	*Processed raw anomalous data from diffraction images in ASCII format*
Experimental factors	*Data from protein crystals subjected to heavy atom derivatization*
Experimental features	*Crystals of periaxin and gliomedin were derivatized with heavy atoms, and anomalous diffraction datasets were collected using synchrotron radiation.*
Data source location	*BESSY, Berlin, Germany*
Data accessibility	*The derivative data are presented in this article and available as*Supplementary material.

**Value of the data**

• Structure solution for periaxin and gliomedin can be reproduced.
• Heavy atom binding to proteins can be understood.
• Derivatization conditions for protein crystals can be optimized.
• Different experimental phasing approaches can be employed.
• The strength of the anomalous signal and diffraction intensity can be studied.

## 1 Data

We recently solved the crystal structures of periaxin and gliomedin [1], [2], and the native crystal data were submitted to the PDB. Here, we include all original unpublished native and derivative datasets used for experimental phasing of the crystal structures, as well as Supplementary datasets with different levels of anomalous signal. The crystallographic data are in Supplementary material, in the output format of the XDS data processing program [3], as this data format allows a variety of further workflows in different software, either directly or after format conversion. In addition, a table of all data processing statistics is given, as are graphs indicating the level and quality of the anomalous signal and diffraction intensity in the different datasets.

## 2 Experimental design, materials, and methods

The preparation of recombinant protein and crystallization for the periaxin PDZ (post-synaptic density-95, discs large, zona occludens-1)-like domain and the gliomedin olfactomedin (OLF) domain have been described [4], [5]. Crystals of the periaxin PDZ-like domain were obtained in 30% polyethylene glycol (PEG) 2000 monomethyl ether, 0.1 M KBr at +4 °C, and tungsten derivatization was completed by soaking the crystals in 5 mM (NH_4_)_2_WS_4_ for 2 days, while Xe-derivatized crystals were prepared at the beamline, by incubating fresh crystals in a Xe chamber at 200 psi for either 2 or 8 min. A gliomedin OLF domain crystal, grown in 0.1 M CHES (pH 9.5) containing 20% PEG 8000, was derivatized with platinum by soaking in 5 mM K_2_PtCl_4_ at room temperature.

All diffraction data were collected on the synchrotron radiation beamline 14.1 at BESSY (Berlin) and processed with XDS [3]. Data processing statistics are given in Table 1. The final crystal structures have been published elsewhere [1], [2].

**Table 1:** Crystallographic data processing statistics.

Protein	Periaxin						Gliomedin
Dataset	Native^a^	Xe 2 min	Xe 8 min^a^	W peak^a^	W inflection	W remote	Pt^a^
Wavelength (Å)	0.918	1.700	1.700	1.2150	1.2155	1.2114	1.071
Space group	P3_2_21						P2_1_
Unit cell							
*a* (Å)	77.28	77.54	77.24	77.68	77.87	77.75	45.51
*b* (Å)	77.28	77.54	77.24	77.68	77.87	77.75	100.65
*c* (Å)	80.48	81.49	81.32	80.14	80.30	79.99	59.27
α (°)	90	90	90	90	90	90	90
β (°)	90	90	90	90	90	90	90.050
γ (°)	120	120	120	120	120	120	90
Resolution range (Å)	50-3.20 (3.28-3.20)	50-3.10 (3.30-3.10)	50-3.30 (3.40-3.30)	50-3.30 (3.40-3.30)	50-3.50 (3.60-3.50)	50-3.71 (3.8-3.71)	50-2.01 (2.06-2.01)
Completeness (%)	99.0 (100)	100 (100)	99.9 (99.9)	99.8 (99.2)	99.8 (99.0)	99.9 (100)	90.4 (89.5)
Redundancy	5.7 (5.8)	14.3 (14.2)	13.8 (13.4)	7.2 (7.5)	14.5 (14.7)	14.3 (14.2)	1.5 (1.5)
<I/σI>	15.1 (2.1)	13.3 (1.1)	11.4 (1.3)	12.5 (1.4)	14.7 (1.5)	11.2 (0.6)	10.4 (2.6)
R_meas_ (%)	10.0 (105.1)	20.6 (273.5)	20.5 (229.2)	10.4 (157.6)	13.8 (209.7)	15.7 (592.9)	6.0 (44.9)
CC_1/2_ (%)	99.9 (60.2)	99.9 (41.6)	99.9 (48.0)	99.9 (43.4)	100 (44.3)	100.0 (6.4)	99.7 (82.7)

a Processing statistics were also reported in the original publications [1], [2], and they are shown to allow direct comparison to the datasets presented here. However, all the original datasets in the table are for the first time made public in this article.

The periaxin structure can be solved using a MIRAS (multiple isomorphous replacement and anomalous scattering) approach with the peak wavelength data for tungsten and the 8-min xenon dataset, in phenix.autosol [6]. In addition, data for the tungsten derivative were collected at the inflection point and at a high-energy remote wavelength, and for xenon, a dataset was also collected after 2-min derivatization.

The gliomedin structure can be solved using SAD (single-wavelength anomalous dispersion) from the attached single dataset of a platinum derivative crystal, for example with programs in the Auto-Rickshaw pipeline [7].

**Fig. 1:**
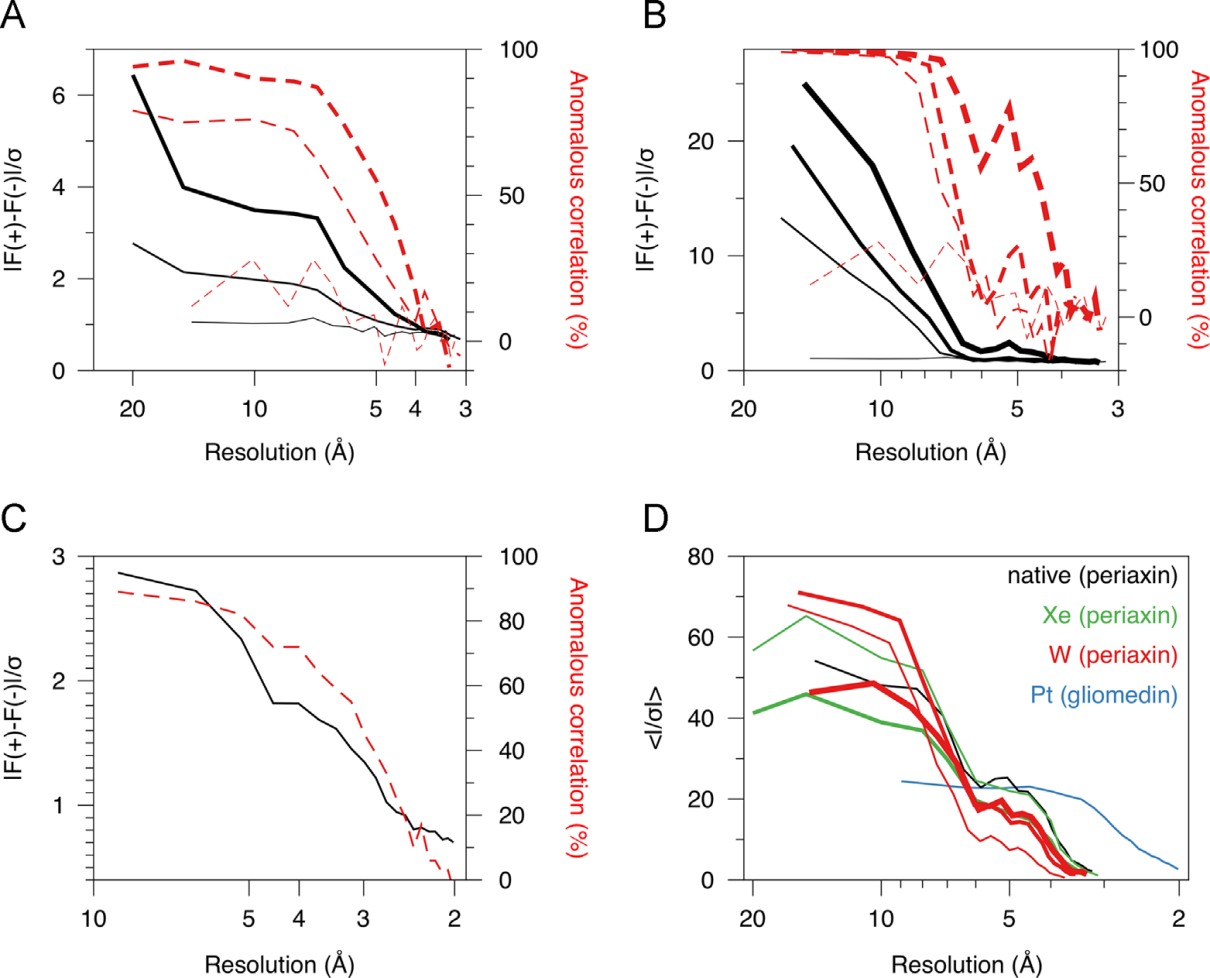
Comparison of anomalous signals in the datasets. The resolution on the X-axis corresponds always to the high-resolution limit of the shell in question. A. Anomalous signal in the Xe-treated crystals. Native data, thin lines; 2-min derivatization, medium lines; 8-min derivatization, thick lines. The anomalous signal (black) and anomalous correlation (red) are as given by the XDS package. B. Anomalous signal in the W-derivatized crystal. From thickest to thinnest: peak wavelength, inflection point, remote energy, native data. C. Anomalous signal in the Pt derivative of gliomedin. D. Diffraction data intensity of all datasets. See inset for color code. For the Xe data, the 2-min (thin) and 8-min (thick) data are plotted. For the W data, line thicknesses are the same as in (B). (For interpretation of the references to color in this figure legend, the reader is referred to the web version of this article.)

Anomalous signals of all the reported datasets are compared in Fig. 1. For xenon derivatization, the signal is much higher with the longer incubation in the pressure chamber. The anomalous signal in different datasets of the tungsten derivative also indicates expected behavior, with the highest signal at the peak wavelength. It should be possible to solve the structure with these data and a MAD (multi-wavelength anomalous dispersion) approach, too.

## Appendix A Supplementary material

The supplementary file archive contains the following crystallographic dataset files, which correspond to the data listed in Table 1, as reflected by their nomenclature:

XDS_ASCII-gldn-pt.HKL

XDS_ASCII-prx-nat.HKL

XDS_ASCII-prx-W-inf.HKL

XDS_ASCII-prx-W-peak.HKL

XDS_ASCII-prx-W-rem.HKL

XDS_ASCII-prx-xe-2min.HKL

XDS_ASCII-prx-xe-8min.HKL
