# Supplementary material for: Crystallographic anomalous diffraction data for the experimental phasing of two myelin proteins, gliomedin and periaxin
Source: Data Brief. 2017 Mar 6;11:552–6. doi: 10.1016/j.dib.2017.02.049 (PMC5567927; doi:10.1016/j.dib.2017.02.049)
Supplement: Supplementary file 1 — Supplementary material [file mmc1.docx]

Confict of interest: none.
